# Commentary: Direct estimation of central aortic pressure from measured or quantified mean and diastolic brachial blood pressure: agreement with invasive records

**DOI:** 10.3389/fcvm.2023.1295467

**Published:** 2023-12-19

**Authors:** Denis Chemla, Mathieu Jozwiak

**Affiliations:** ^1^ INSERM UMRS 999, Hôpital Marie Lannelongue, Le Plessis-Robinson, France; ^2^ Service de Médecine Intensive Réanimation CHU de Nice, Nice, France; ^3^ UR2CA, Unité de Recherche Clinique Côte D'Azur, Université Côte D’Azur, Nice, France

**Keywords:** aortic pressure, brachial blood pressure, non-invasive records, arterial tonometry, physiological measurements, human physiology

A Commentary on Direct estimation of central aortic pressure from measured or quantified mean and diastolic brachial blood pressure: agreement with invasive records By Bia D, Salazar F, Cinca L, Gutierrez M, Facta A, Zócalo Y, Diaz A (2023). Front. Cardiovasc. Med. 10: doi: 10.3389/fcvm.2023.1207069

## Introduction

Our group recently proposed a novel method for estimating central aortic systolic blood pressure (aoSBP) known as Direct Central Blood Pressure (DCBP) estimation (1). This method relies solely on mean blood pressure (MBP) and diastolic blood pressure (DBP) values. It was a proof of concept and invasive validation study, with clear-cut preliminary results that could have significant clinical implications. We read with great interest the article by Bia et al., which investigated the agreement between invasive aoSBP (measured using fluid-filled catheters) and non-invasively obtained DCBP at the brachial artery level in eighty-nine subjects (using oscillometry/plethysmography with the Mobil-O-Graph device) (2). The authors conclude that the utility of the DCBP method depends significantly on both the approach used to estimate brachial mean blood pressure (bMBP) and the aoSBP level. We appreciate the authors’ interest in our proposal (1) and commend them for their well-executed study and interesting results. In this commentary, we will emphasize the rationale for DCBP, discuss the way MBP is estimated in large arteries, explore the strengths and limitations of the current study (2), and suggest potential areas for further research.

## Direct central blood pressure (DCBP) estimation

The DCBP is calculated using this straightforward equation:

$$\mathrm{DCBP}\;=\;\mathrm{MB}P^{2}/\mathrm{DBP}$$

The rationale is as follows: (i) aortic MBP is reliably calculated by taking the square root of the product of aoSBP and aortic DBP (geometric mean) (3); and (ii) it is generally assumed that MBP and DBP remain constant along the arterial tree (4, 5).

Our study (1) achieved high accuracy and good precision of DCBP through the use of intra-arterial BP measurements. Ideally, when applying DCBP in clinical practice, minimizing errors in BP measurements must be sought, as is the case with all devices aiming to non-invasively estimate aoSBP. While non-invasive BP measurement errors are unavoidable, the mathematical formalism of DCBP implies that an overestimation of both MBP and DBP tends to mutually cancel out (as does an underestimation of both) (1). This explains why, in the Bia et al. study (2), non-invasive DCBP predicted aoSBP with a 1% mean error (137 vs. 135 mmHg), while bMBP and bDBP were 7% and 14% higher than their invasive central values (1.07^2^/1.14 = 1.01).

## Estimation of MBP in large arteries

MBP represents the time-averaged arterial blood pressure throughout the cardiac cycle. Invasive studies have revealed that MBP can be estimated by adding a fraction of pulse pressure to DBP, known as the form factor (6, 7), as it varies depending on the pulse wave's form. In adults, the mean form factor value ranges from 0.41 to 0.45 in the central aorta (6, 8, 9), 0.37 to 0.40 at the brachial artery level (10, 11), and 0.31 to 0.36 at the radial artery level (11–13). This decrease in form factor reflects the central-to-peripheral systolic blood pressure amplification phenomenon, whose extent can vary with arterial site, sex, age, BP level, and physiological or pathological conditions (4, 5). At the aortic level, five different equations relying on aortic SBP and DBP have been invasively validated (3, 8, 9, 14, 15), all providing essentially similar and physiologically relevant central MBP values. Finally, accurate invasive BP monitoring with an arterial catheter demands a deep understanding of measurement principles, including transducer leveling and zeroing, system damping, BP waveform quality, and precise reading interpretation (16).

As far as the non-invasive estimation of MBP is concerned, using current cuff-based methods presents challenges, as many do not display bMBP, and the best empirical formula for estimating it from SBP and DBP remains a subject of ongoing discussion. A 0.40 form factor value is generally favored (10), although no universal value would achieve a highly precise estimation of bMBP given interindividual variability (11).

## Strengths and limitations of the current study

One strength of the study (2) is its meticulous assessment of the dependency of DCBP (as an estimate of aoSBP) on the approach used to estimate MBP, considering that MBP is squared in the DCBP formula. A notable novelty of the study is its exploration of DCBP's accuracy and precision in relation to prevailing aoSBP levels. The pressure-dependent errors documented by the authors at the brachial level (2) have not been observed at the aortic or radial levels (1, 17), suggesting the necessity for additional explanation and confirmation. The authors have addressed some limitations of the study, but there are a few other important ones that also require consideration. Firstly, the patients exhibited similar measured invasive aortic and brachial SBP. However, the study's findings might not be applicable to patients showing marked pressure wave amplification phenomenon at the brachial level. Secondly, the results strictly apply to the non-invasive device under study. Our previous sensitivity analysis (1) underscores the potential challenge of low accuracy in DCBP, especially when dealing with a non-invasive device that introduces opposing measurement errors, such as coupled underestimation of MBP alongside overestimation of DBP, or vice versa. Overall, this highlights the need for improving non-invasive MBP estimation, and we concur with the authors in emphasizing the importance of cuff-based method manufacturers (e.g., oscillometric devices) providing bMBP values calculated by their own internal algorithms.

## Further research

At that point, one may ask how DCBP compares to non-invasive tonometry-derived aoSBP? To answer this question, our group recently conducted a non-invasive study on 160 patients using radial applanation tonometry with the SphygmoCor calibrated on brachial SBP/DBP (17). The time-averaged MBP was calculated from the radial pulse waveform. The (DCBP—aoSBP) error was −1.4 ± 4.9 mmHg (−1.1 ± 3.9%), showing no influence by the mean. This suggests that using radial tonometry, DCBP and non-invasive aoSBP may be interchangeable, thus rendering a generalized transfer function unnecessary. If confirmed by other studies, this finding could have significant clinical implications.

## Conclusion

We extend our thanks and congratulations to the authors for conducting a comprehensive study on the strengths and limitations of our DCBP formula. While the path to brachial-cuff-derived estimation of aoSBP may seem long, we hope this discussion on DCBP will also stimulate new research to enhance the accuracy of peripheral MBP estimation.
